# Impact of genomics on the field of probiotic research: historical perspectives to modern paradigms

**DOI:** 10.1007/s10482-014-0171-y

**Published:** 2014-04-20

**Authors:** Brant R. Johnson, Todd R. Klaenhammer

**Affiliations:** 1 Department of Microbiology, North Carolina State University, Raleigh, NC USA; 2 Department of Food, Bioprocessing, and Nutrition Science, North Carolina State University, Raleigh, NC USA

**Keywords:** Probiotic, Lactic acid bacteria, Fermentation, Genomics, Lactobacilli, Bifidobacteria

## Abstract

For thousands of years, humans have safely consumed microorganisms through fermented foods. Many of these bacteria are considered probiotics, which act through diverse mechanisms to confer a health benefit to the host. However, it was not until the availability of whole-genome sequencing and the era of genomics that mechanisms of probiotic efficacy could be discovered. In this review, we explore the history of the probiotic concept and the current standard of integrated genomic techniques to discern the complex, beneficial relationships between probiotic microbes and their hosts.

## Introduction

### History of probiotic bacteria and the probiotic concept

A multitude of autochthonous (naturally occurring) commensal bacterial species inhabit the mucosal surfaces of the gastro-intestinal tract (GIT), as well as those of the nose, mouth and vagina. It has long been held that the consumption of allochthonous (transient) beneficial bacteria, either through food products or supplements, has a positive influence on general health and well-being of the host via commensal interactions with the GIT immune system and resident microbiota. These beneficial microorganisms, known as probiotics, are defined by the World Health Organization as “live microorganisms, which when administered in adequate amounts, confer a health benefit upon the host” (FAO/WHO 2002). Over the past four decades, there has been substantial research in the field of probiotics and, more specifically, into the mechanism of probiotic action within the host. However, the probiotic concept is not novel to the twentieth century and twenty-first centuries.

For millennia, humans have consumed microorganisms via fermented foods, which served to prevent putrefaction as well as increase sensory aspects in the food. Some of the first fermentations were likely the result of serendipitous contaminations in favourable environments resulting in soured milk products such as kefir, leben, koumiss, yogurt and sour cream—products that are still consumed worldwide (Hosono 1992). Furthermore, through the continued practice of milk souring along with back slopping techniques, humans inadvertently aided in the domestication of certain microorganisms to diverse food environments over time (Douglas and Klaenhammer 2010). Not only were these products safe to consume, fermented dairy foods were culturally significant, as evidenced by their mention in the Bible and early sacred Hindu texts, as well as therapeutically consumed (Hosono 1992; Bibel 1988; Shortt 1999).

**Fig. 1:**
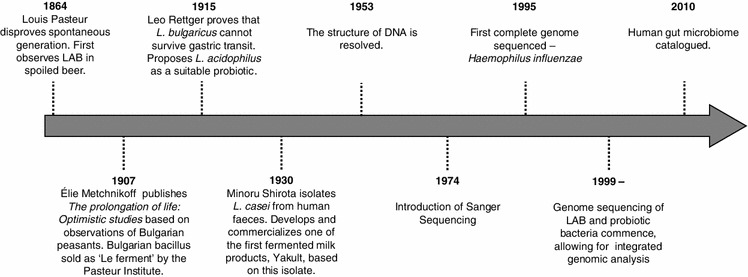
Seminal milestones contributing to the functional characterization of probiotic lactic acid bacteria

In the late nineteenth century, French biochemist Louis Pasteur premiered significant discoveries leading to a greater scientific understanding of fermentation (Fig. 1). Upon studying wine and beer fermentations, Pasteur demonstrated that fermentation reactions are carried out by microorganisms. Furthermore, he established that the growth of these microbes is not a product of spontaneous generation, as was the prevailing scientific and cultural consensus, but is instead due to biogenesis, which posits that all living things come only from other living things. On the foundation of Pasteur’s research, Russian Nobel laureate, Élie Metchnikoff first popularized the concept of probiotics around the turn of the twentieth century. In his book, *The Prolongation of Life: Optimistic Studies*, Metchnikoff (1907) proposed that putrefaction in the intestines correlated with shortened life expectancy. Reconciling long-held observations involving lactic acid food fermentations with microbial feeding studies in animals and humans, Metchnikoff proposed that lactic acid-producing microorganisms may act as anti-putrefactive agents in the gastrointestinal tract when consumed. In fact, he hypothesized that by transforming the “wild population of the intestine into a cultured population… the pathological symptoms may be removed from old age, and… in all probability, the duration of the life of man may be considerably increased” (Metchnikoff 1907). His theory was bolstered upon observing a higher prevalence of centenarians in Bulgaria, a region known for the consumption of soured milk. Michel Cohendy, a colleague at the Pasteur Institute, provided experimental data to support Metchnikoff’s hypothesis. In two feeding trials of human subjects, Cohendy found that the Bulgarian bacillus (now known as *Lactobacillus delbrueckii* subsp. *bulgaricus*) was recoverable from faeces; reduced the prevalence of putrefactive toxins; and aided in the treatment of colitis following transplantation to the large intestine (Cohendy 1906a, b). The aforementioned studies on *L. bulgaricus* enthralled the health-conscious society of Europe in the early 20^th^ century and soon the Pasteur Institute of Paris began selling the *Lactobacillus* under the label of “Le Ferment” (Shortt 1999; Bibel 1988).

Despite the promising observations made by Metchnikoff and colleagues at the genesis of the probiotic concept, there was still meager scientific evidence suggesting any definitive probiotic strains or their purported effector mechanisms. In fact, Leo Rettger and coworkers at Yale University found that *L. bulgaricus* could not survive gastric passage to colonize the small intestine (Rettger 1915). This study called into question which strain(s) may have been present in the original therapeutic administration studies performed by Cohendy, and subsequently sold as “Le Ferment.” Instead, *Lactobacillus acidophilus* was touted to be a more suitable candidate for therapeutic applications because of its ability to survive gastric passage and transform the intestinal flora in conditions of lactose and dextrin supplementation (Rettger and Cheplin 1921). It is based on these seminal studies that the foundation of therapeutic treatment with *L. acidophilus* originated. However, even rigorous studies such as these were limited by the techniques and technologies of their time. *L. acidophilus* could not be distinguished from other aciduric commensal lactobacilli, such as *Lactobacillus gasseri*, until electrophoretic DNA–DNA hybridization studies on *Lactobacillus* lactate dehydrogenase enzymes were performed in the 1970s (Gasser 1970; Gasser et al. 1970). Therefore, it is unknown whether the cultures administered during these studies were indeed pure *L. acidophilus*, or mixed culture with *L. acidophilus*, *L. gasseri* and other aciduric lactobacilli.

After examining the burgeoning experimental evidence of probiotic bacteria, a Japanese physician named Minoru Shirota sought to isolate a human-derived strain of *Lactobacillus* for therapeutic application. And thus, in 1930, Shirota selected a species of *Lactobacillus* (now known as *Lactobacillus casei* Shirota) from human faeces that could survive passage through the GIT (Shortt 1999). From this culture, Shirota developed and commercialized one of the first fermented milk products, Yakult (Shortt 1999). Not only was this a major advancement for the commercial dairy industry, but one of the first products to deliver a pure, defined strain-cultured product. Yakult remains a staple product in Japanese, Korean, Australian and European markets. Since then, there has been a massive expansion of the functional food market, especially in fermented dairy products containing probiotic bacteria (Sanders and in’t Huis-Veld 1999). In fact, a recent global market analysis on probiotics revealed a 7 % annual growth during the 2012 fiscal year, with a forecast of $48 billion in earnings within the next 5 years (Global Industry Analysis Report 2012). Furthermore, probiotics are expanding from functional food markets to pharmaceutical, therapeutic markets. This market increase correlates to the advancements of the scientific and regulatory aspects of probiotic mechanisms and delivery (Foligne et al. 2013). Considering that there are still a great number of scientific questions to explore concerning probiotic activities and interactions in the GIT, there remains a bright future for the field of probiotic research and the market thereof.

### Modern use of probiotic bacteria

Despite the long, storied history of probiotic discovery and therapeutic application, resounding clinical and experimental evidence for the use of probiotic bacteria has only recently come to a head (Table 1). One prominent example is the use of probiotics to treat functional gastrointestinal disorders (FGID). For many FGID, such as irritable bowel syndrome (IBS), there are few pharmacological treatment options due to low efficacy and serious side effects (Shen and Nahas 2009). Furthermore, IBS is quite common and is thought to be caused by changes in the gastrointestinal microbiome (Porter et al. 2011). Recently, a systematic review of successful clinical interventions using probiotics to treat various FGID has been compiled as a reference for clinicians to make evidence-based treatment decisions (Hungin et al. 2013). This systematic analysis reflects a notable caveat that must be made in probiotic research; namely, that probiotic activities are strain-specific (Hungin et al. 2013; Sanders et al. 2013). Because evidence clearly suggests not only the efficacy of probiotic therapy, but also the importance of understanding each strain, the paradigm of probiotic research is rightfully shifting towards understanding the mechanistic action of each specific strain.

**Table 1:** Roles and benefits of probiotic bacteria in the GIT

Probiotic role/benefit	Reference
Protection against infection	Corr and O’Neill (2009)
Symptom relief from irritable bowel syndrome	Hungin et al. (2013)
Lactose digestion for lactose-intolerant individuals	Mattila-Sandholm et al. (1999)
Lowered incidence of diarrhea	Leyer et al. (2009)
Lowered risk of antibiotic-associated diarrhea	Gao et al. (2010)
Lowered risk of *C. dificile*-associated diarrhea	Plummer et al. (2004), Gao et al. (2010)
Reduction in intestinal bloating	Ringel-Kulka et al. (2011)
Abdominal pain analgesic (via μ-opiod and cannabinoid receptors)	Rousseaux et al. (2007)
Lowered levels of cold and influenza-like symptoms in children	Leyer et al. (2009)
Antimicrobial activity	Ryan et al. (2009)
Competitive exclusion of pathogens	Lee et al. (2003)
Inhibition of *H. pylori* growth	Ushiyama et al. (2003); Fujimura et al. (2012)
Reduced incidence of necrotizing enterocolitis	Deshpande et al. (2010)
Prevention of upper respiratory infections	Hao et al. (2011)
Immune tolerance	van Baarlen et al. (2009)
Reduction in colorectal cancer biomarkers	Rafter et al. (2007)
Return to pre-antibiotic baseline flora	Engelbrektson et al. (2009)
Epithelial barrier function	Mennigen and Bruewer (2009)
Increased natural killer cell activity	Takeda and Okumura (2007)
Increased humoral immunity via secretion of IgA	Viljanen et al. (2005)
Lowered blood cholesterol levels	Ataie-Jafari et al. (2009)
Reduction in irritable bowel disease symptoms	MacFarlane et al. (2009)
Delivery of therapeutics	Wells and Mercenier (2008)

Modified from O’Flaherty and Klaenhammer (2010a)

Among the most studied species of probiotic bacteria are those from the genera *Lactobacillus* and *Bifidobacterium* (Table 2). The genus *Lactobacillus* is comprised of a diverse clade of Gram-positive, anaerobic/microaerophilic, non-sporulating, low G + C content lactic acid bacteria (LAB) belonging to the phylum *Firmicutes* (Pot et al. 1994). Biochemically, they are strictly fermentative; sugar fermentations result in either the sole production of lactic acid, or the production of lactic acid in conjunction with smaller amounts of carbon dioxide and ethanol/acetic acid (Hammes and Vogel 1995; Pot et al. 1994). Lactobacilli inhabit diverse ecological niches including the GIT of humans and animals, as well as vegetable, plant and dairy food environments. While *Lactobacillus* species are not dominant members of the colonic microbiotia, many are probiotic because of their ability to survive in the less-diverse small intestine. Members of the genus *Bifidobacterium*, of the phylum *Actinobacteria,* are Gram-positive, non-motile, anaerobic bacteria, with low levels of genomic and phylogenetic diversity (Ventura et al. 2006). They were originally isolated from the faeces of breast-fed infants (Tissier 1900) and nearly 50 species isolated from the GIT of humans animals and insects have since been classified (Velez et al. 2007). In fact, bifidobacteria are among the most prominent commensal bacteria found in the human colon and dominate the developing microbiome in breast-fed infants (Turroni et al. 2008; Favier et al. 2002).

**Table 2:** Common probiotic *Lactobacillus* sp. and *Bifidobacterium* sp.

Probiotic (strain designation)	Genome sequence reference (accession number)
*Lactobacillus*	
*L. acidophilus* (NCFM, La-1)	Altermann et al. (2005) (NC_006814.3)
*L. casei* (BL23)	Maze et al. (2010) (NC_010999.1)
*L. johnsonii* (NCC 533)	Pridmore et al. (2004) (NC_ 005632.1)
*L. plantarum* (JDM1)	Zhang et al. (2009) (NC_012984.1)
*L. reuteri* (SD2112)	(NC_015697.1)
*L. rhamnosus* (GG)	Kankainen et al. (2009) (NC_013198.1)
*L. salivarius* (UCC118)	Claesson et al. (2006) (NC_007929.1)
*L. bulgaricus* (ATCC 11842)	van de Guchte et al. (2006) (NC_008054.1)
*Bifidobacterium*	
*B. animalis* subsp. *lactis* (B1-04)	Barrangou et al. (2009) (NC_012814.1)
*B. breve* (UCC2003)	O’Connell Motherway et al. (2011) (NC_020517.1)
*B. longum* (NCC 2705)	Schell et al. (2002) (NC_004307.2)

**Fig. 2:**
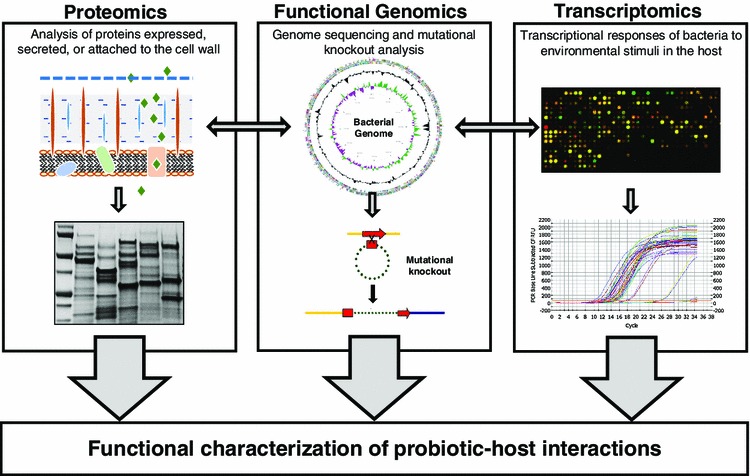
With the advent of genome sequencing, integrated genomic techniques including proteomics, transcriptomics and functional genomics have collectively characterized the mechanism of probiotic host-interactions. These analyses rely on access to annotated sequence data from whole genome sequencing. Genetic systems for deletions and mutational knockouts allow for phenotyping specific genetic loci. Proteomic approaches involve the characterization of proteins expressed, secreted, and/or attached to the cell wall. In this way, proteins are isolated, characterized by mass spectrometry, and mapped back to the proteome and corresponding genome for functional analysis. Finally, transcriptomic profiling using DNA microarrays, RNA sequencing, and RT-qPCR can measure the transcriptional responses of both bacteria and host cells in response to one another, via measurement of mRNA

Since the resolution of the first bacterial genome sequence (*Haemophilus influenzae*), an exponential advancement in sequencing processing, genome assembly and annotation technologies, at increasingly economical pricing, has yielded well over a thousand publicly available genomes (Fleischmann et al. 1995; Lagesen et al. 2010). Notably, many of these genomes are derived from lactic acid bacteria used as probiotics or starter cultures for food fermentations (Klaenhammer et al. 2002; Lukjancenko et al. 2012). Access to these data has revolutionized the molecular view of probiotic bacteria, as well as the way research questions related to probiotic mechanisms are formulated. Specifically, advancements in genomic tools including functional genomics, transcriptomics, proteomics and secretomics, have hastened research deciphering the interactions between probiotics and the GIT (Fig. 2). These techniques are being used to bridge the mechanistic gap between what has been seen clinically and anecdotally for hundreds of years.

### Characterizing probiotic mechanisms using genomic tools

**Fig. 3:**
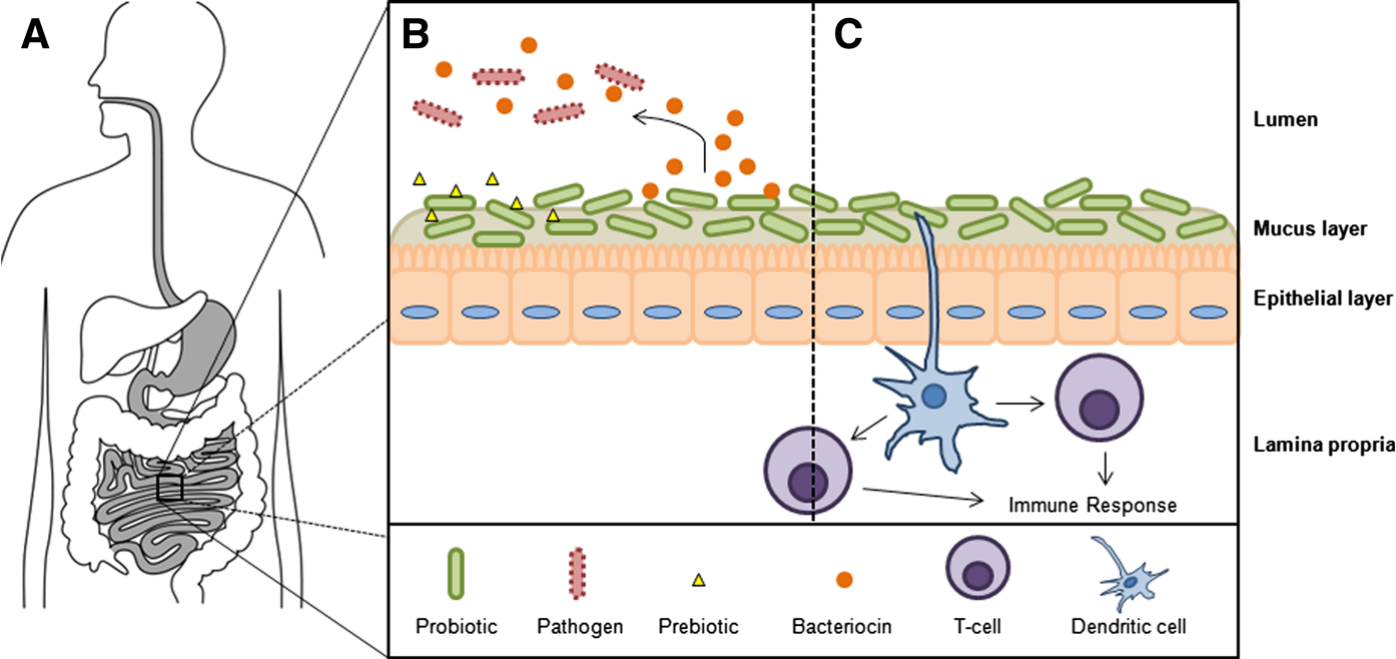
**a** Probiotic microbes delivered orally must survive varying environments encountered through gastrointestinal transit, including acidic gastric juices (pH ~2) in the stomach, and bile in the small intestines. **b** At the intestinal epithelia, probiotics have been reported to adhere in high numbers, leading to competitive exclusion of pathogens. The growth of certain probiotics can be stimulated by the presence of complex prebiotic oligosaccharides. Additionally, some probiotics produce bacteriocins and other antimicrobial agents which may antagonize pathogens in the lumen. **c** Probiotics bound in the mucus and epithelial layers are proximal to dendritic cells of the mucosal immune system, leading to immunomodulation

Referencing the genome sequences of probiotic bacteria, the mechanism and interaction of probiotics with the host GIT are being discovered through the integration of functional genomic techniques. Within this context, there are three points of focus relating to probiotic action: (i) survival through gastrointestinal transit and adhesion to intestinal epithelia; (ii) competitive exclusion and antimicrobial activity; and (iii) modulation of the host GIT immune system (Fig. 3).

### Survival in and adhesion to the GIT

One of the most essential qualities of a probiotic microorganism is the ability to survive the varied environments of the GIT (Fig. 3a). The probiotic must be able to adapt to acidic gastric juices and bile in the small intestine. Like many aciduric bacteria, the lipid membranes of lactobacilli exposed to acid and bile are altered in order to increase survival. The lipid membrane of *Lactobacillus casei* demonstrated a marked increase of mono-unsaturated fatty acids in response to acidification (Fozo et al. 2004). Similarly, the lipid membrane of *Lactobacillus reuteri* exposed to bile salts and cholesterol increased the number of mono-unsaturated fatty acids compared to saturated fatty acids (Taranto et al. 2003). Considering these observations, a recent study using the probiotic *Lactobacillus rhamnosus* GG found that an exogenous oleic acid [C18:1 (cis-9)] source significantly increased acid survival by incorporating the oleic acid into the membrane, which is reduced to stearic acid (C18:0) in the acidified environment (Corcoran et al. 2007). Aside from the biochemical changes to the lipid membranes, the *Lactobacillus* species have global transcriptional responses to these stressors, usually through two-component regulatory systems (2CRS; Lebeer et al. 2008b). Numerous transcriptomic analyses have been used in lactobacilli to identify differentially expressed genes, such as those corresponding to 2CRS, surface proteins and proton efflux systems, in response to gastric acid stress (Azcarate-Peril et al. 2005; Pieterse et al. 2005) and bile stress (Bron et al. 2006; Pfeiler et al. 2007). Bacteria quickly sense and respond to changing environmental conditions via 2CRS through the sensing domains of a transmembrane histidine protein kinase (HPK). Upon receiving the environmental signal, the HPK is activated to autophosphorylate a specific histidine residue which is transferred to the regulatory domain of the response regulator (RR), a DNA-binding transcriptional regulator. Therefore, 2CRS can be predicted from bacterial genome sequence annotations based on the presence of putative HPK and RR in close proximity to one another (Altermann et al. 2005; Morita et al. 2009). In *L. acidophilus* NCFM, a gene (*lba1524*) encoding a functional HPK was knocked out, resulting in a mutant with increased sensitivity to acid stress compared to the parent strain. Furthermore, transcriptomic analysis via DNA microarray comparing the *lba1524* mutant to wild-type demonstrated an impact on 80 genes (Azcarate-Peril et al. 2005). Notably, one upregulated gene in the HPK mutant was the LuxS homolog of the autoinducer-2 quorum sensing compound, important for survival in gastric juices and adhesion to intestinal epithelial cell lines (Lebeer et al. 2008a; Buck et al. 2009).

The response of lactobacilli to bile salts has also been measured through microarray analysis. In *Lactobacillus plantarum* a DNA-microarray was performed after exposure to porcine bile, resulting in the identification of bile response genes encoding stress response proteins, cell envelope proteins and an F_0_F_1_ ATPase (Bron et al. 2006). A similar transcriptomic profiling of *L. acidophilus* revealed multiple genes involved in bile tolerance, including a 2CRS and multi-drug resistance (MDR) transporter efflux pumps (Pfeiler et al. 2007). Mutants with insertionally inactivated genes for the bile inducible 2CRS HPK and RR were more sensitive to bile compared to parent strains, confirming their role in bile tolerance (Pfeiler et al. 2007). A recent comparative proteomic analysis on bile sensitive and bile tolerant strains of *L. plantarum* corroborated these transcriptomic data and elucidated potential biomarkers for the selection of bile tolerant probiotic strains (Hao et al. 2011). Additionally, the role of efflux pumps and MDR transporters in probiotic bile tolerance are beginning to be recognized. Functional genomic analyses of MDR transporters in probiotic strains of *L. reuterii* and *L. acidophilus* demonstrated roles in bile tolerance (Whitehead et al. 2008; Pfeiler and Klaenhammer 2009). Furthermore, a MDR transporter gene in *Bifidobacterium longum*, *betA* (bile efflux transporter), was recently identified through in silico genome analysis and functionally characterized (Gueimonde et al. 2009). Heterologous expression of *betA* in *Escherichia coli* conferred bile tolerance through active efflux of bile salts.

Survivability and enhancement of beneficial microbes in the GIT can be accomplished by providing selectively utilizable carbohydrates, called prebiotics (Roberfroid 2007; Andersen et al. 2013). These carbohydrates, including *β*-galactooligosaccharide (GOS), lactulose, fructo-oligosaccharide and inulin, are resistant to gastric acidity, hydrolysis and gastrointestinal absorption (Roberfroid et al. 2010). As growth substrates, prebiotic carbohydrates are preferentially metabolized by species of health-promoting bacteria. Recently, differential transcriptomic and functional genomic analyses have demonstrated the capabilities of the probiotic bacteria *L. acidophilus* NCFM (Andersen et al. 2012) and *Bifidobacterium lactis* B1-04 (Andersen et al. 2013) to utilize prebiotic oligosaccharides. With these data, novel symbiotic formulations of corresponding prebiotics for *L. acidophilus* and *B. lactis* can be created to aid in the survival and probiotic effectiveness in the host small intestines and colon, respectively. In a similar vein, there is compelling evidence to suggest glycogen metabolism is a colonization factor for probiotic LAB. Glycogen is a large molecular mass, soluble *α*-1,4-linked glucose polymer with numerous *α*-1,6-linked branches. It has multiple physiological functions in various bacteria and has been theorized to function as a carbon capacitor for the regulation of energy flux (Belanger and Hatfull 1999). Recent work by Goh and Klaenhammer (2013) demonstrated the functionality of a putative glycogen metabolism operon found in the genome sequence. Remarkably, through a series of chromosomal deletions and phenotypic assays, glycogen metabolism was found to regulate growth maintenance, bile tolerance and complex carbohydrate utilization in *L. acidophilus* (Goh and Klaenhammer 2013).

**Fig. 4:**
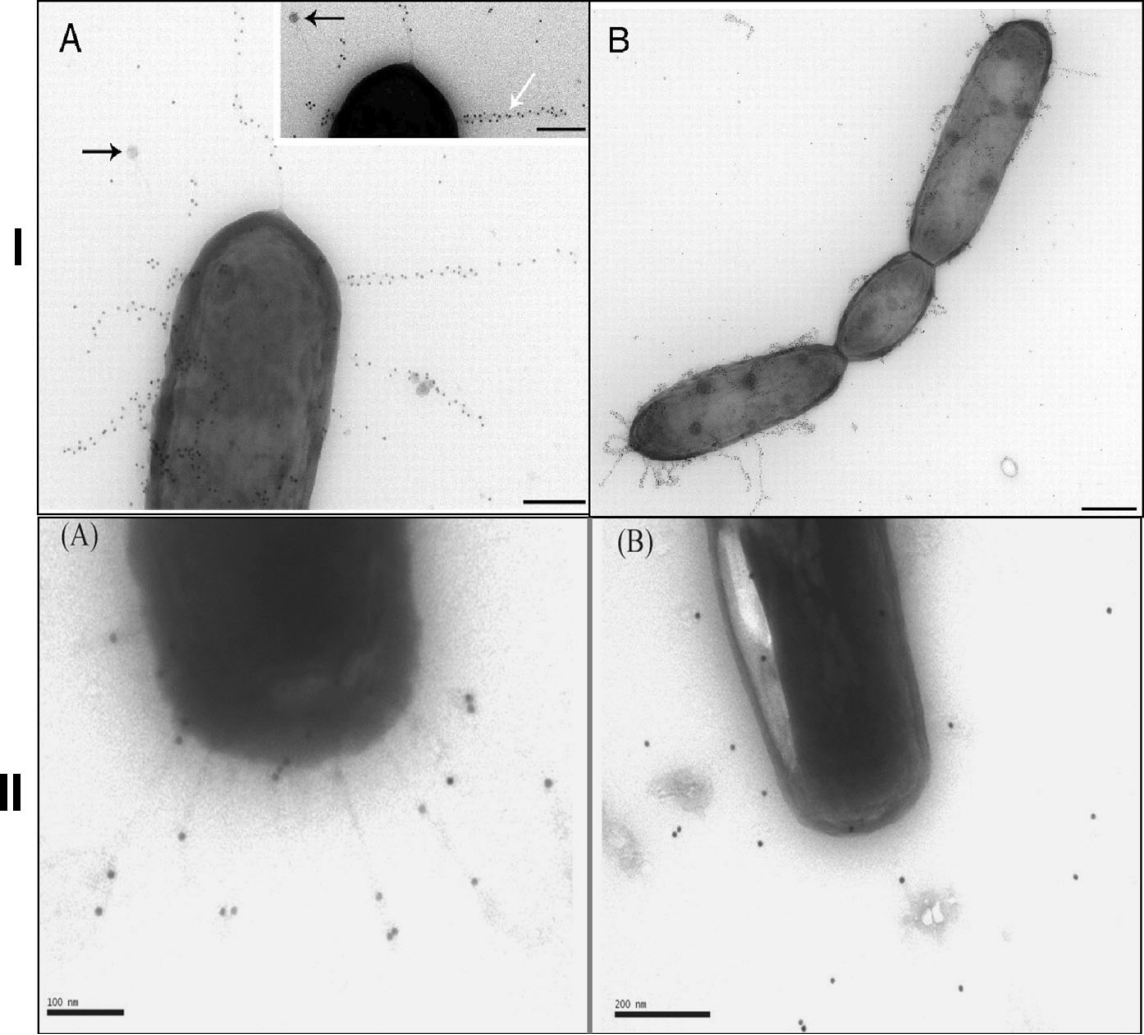
Identification of pili structures in *Lactobacillus rhamnosus* GG (**I**) and *Bifidobacterium breve* UCC2003 (**II**). Images were obtained using transmission electron microscopy on negatively stained, immunogold-labeled anti-*SpaC* pili in *L. rhamnosus* (**I**) and anti-*Flp*_*2003*_ pili in *B. breve* (**II**). Reprinted with permission from Kankainen et al. (2009), copyright © 2009 National Academy of Sciences, USA; and O’Connell Motherway et al. (2011), copyright © 2011 National Academy of Sciences USA

Beyond surviving gastrointestinal transit, a second key factor for probiotic activity is through adhesion to intestinal epithelia of the GIT. Preliminary in vitro studies using Caco-2 human intestinal epithelial cell lines revealed multiple probiotic lactobacilli with adhesive capabilities (Chauviere et al. 1992; Tuomola and Salminen 1998). Notably, there has also been work demonstrating the adhesiveness of *Bifidobacterium* spp. to human intestinal mucus (He et al. 2001). However, access to genome sequence data, paired with integrated genomic techniques, elucidated mediators of probiotic adhesion. The majority of these factors are secreted or attached to the cell wall in a sortase-dependent manner, in order to interface with the intestinal epithelia (reviewed by Velez et al. 2007 Lebeer et al. 2008b). In a study using *L. plantarum* WCFS1, two of these sortase-dependent proteins (SDP) were found to be induced in the murine GIT (Bron et al. 2004a, b). Mutational analysis of one of these genes (*lp_2940*) resulted in a 100- to 1,000-fold decrease in persistence capacity of the *L. plantarum* lp_2940 knockout mutant in a mouse model. In *L. acidophilus* NCFM, in silico genome screening lead to the selection of five putative adhesion cell surface proteins, including a fibronectin binding protein (FbpA), S-layer protein (SlpA), mucin-binding protein (Mub) and two R28 homologues involved in streptococcal adhesion (Buck et al. 2005). Through mutational analysis, FbpA, Mub, and SlpA were all found to contribute to adhesion to Caco-2 epithelial cell lines. Similarly, a stress response protein and an aggregation-promoting factor (both cell surface proteins) were found in later studies to contribute to adherence to Caco-2 cells (O’Flaherty and Klaenhammer 2010b; Goh and Klaenhammer 2010). In *Lactobacillus crispatus* JCM5810, the S-layer protein (CbsA) contains domains that bind to laminin and collagens (Antikainen et al. 2002). Genome screening and secretome analysis of *Lactobacillus salivarius* UCC118 led to the identification of three SDPs with mucus-binding domains. A sortase-deficient strain was created, resulting in significantly reduced adherence to Caco-2 and HT-29 cell lines in vitro (van Pijkeren et al. 2006). Notably, genomic analysis between two strains of *L. rhamnosus* revealed the presence of a genomic island in *L. rhamnosus* GG that contained three secreted, sortase-dependent pilins encoded by *spaCBA* (Kankainen et al. 2009). Immunoblotting and immunogold electron microscopy confirmed the formation of cell wall-bound pili (Fig. 4). Furthermore, mutational analysis of the *spaC* gene abolished the adherence capability of *L. rhamnosus* GG to human intestinal mucus, implicating the role of these unique pili structures in adherence and retention in the GIT. Since this initial report, a type IVb tight adherence (Tad) pilus-encoding gene cluster has been identified in *Bifidobacterium breve* UCC2003 (O’Connell Motherway et al. 2011; Fig. 4). Mutational analysis demonstrated that the Tad gene cluster was essential for colonization in a murine model. Collectively, these data suggest that there are multiple cell surface factors which contribute to probiotic adherence to human intestinal epithelia.

### Competitive exclusion and antimicrobial activity

Another health-promoting aspect of probiotic bacteria is the prevention of pathogenic infection (Fig. 3b). When probiotic lactobacilli are ingested, they temporarily coat the mucosal layer and epithelia of the small intestine (see above) leading to both physical and chemical barriers against harmful bacteria (Servin 2004). Initial studies demonstrated that lactobacilli inhibited adherence of Gram-negative uropathogens when uroepithelial cells were pre-incubated with whole, viable *Lactobacillus* (Chan et al. 1985). Furthermore, in vivo mice models demonstrated that *L. casei* GR1 was capable of preventing urinary tract infections from *E. coli*, *Klebsiella pneumoniae*, and *Pseudomonus aeruginosa* (Reid et al. 1985). In both cases, the mechanism of pathogenic antagonism was due to the ability of lactobacilli to adhere to the urogenital epithelia, thus preventing infection through competitive exclusion of the pathogen. These studies and others suggested that similar competitive exclusion could be possible in the human GIT using probiotic lactobacilli and bifidobacteria. In fact, numerous studies have demonstrated the in vitro inhibition of numerous gastrointestinal pathogens through competitive exclusion of probiotic lactobacilli and bifidobacteria using intestinal cell lines (reviewed by: Servin 2004).

**Fig. 5:**
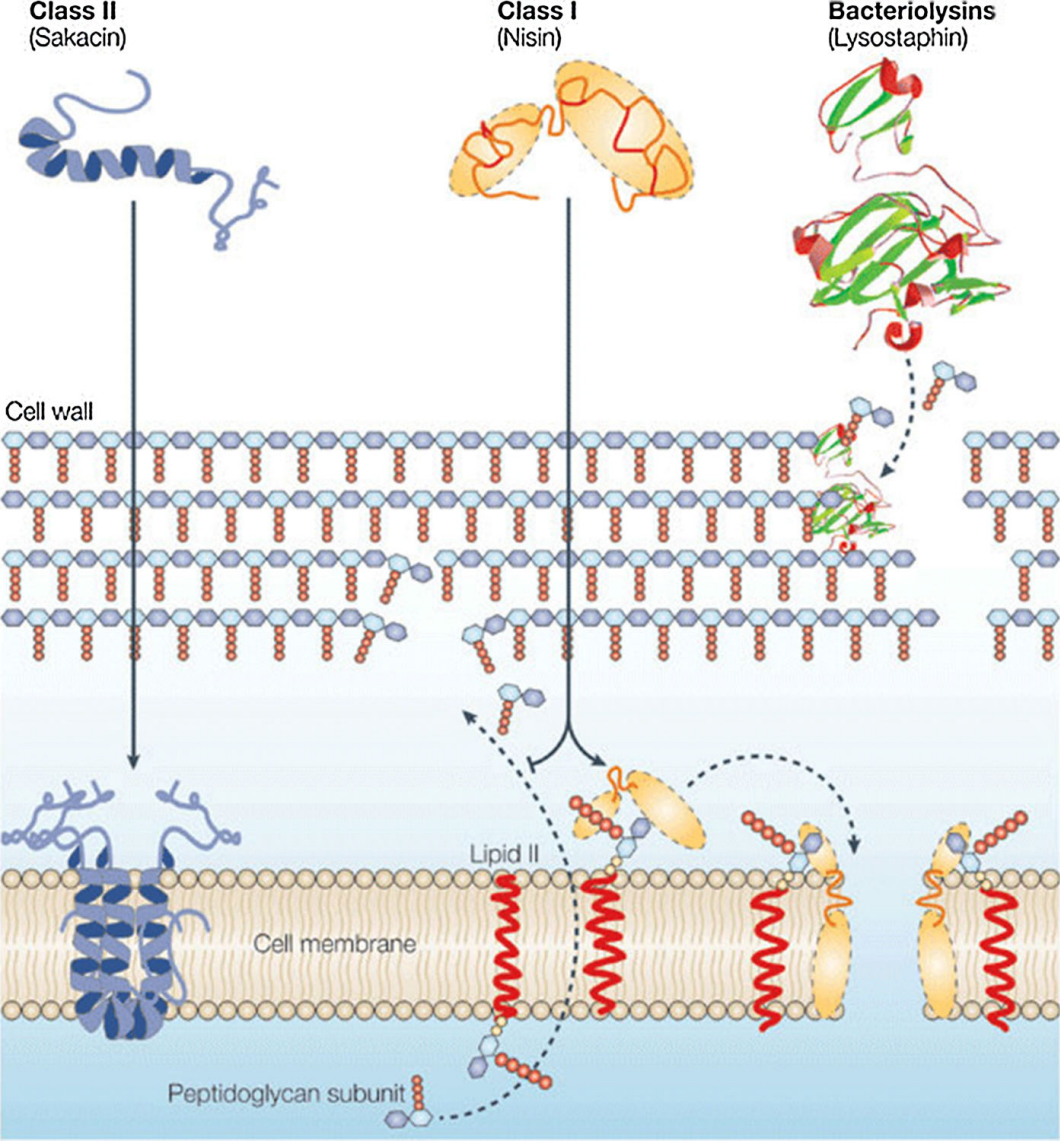
Bacteriocins produced by LAB are grouped into three classes based on structure and function: *class I* (lantibiotics), *class II*, and bacteriolysins. *Class I* lantibiotics, such as nisin, can have two modes of action. First, they bind lipid *II* to prevent peptidoglycan subunit transport, disrupting peptidoglycan synthesis and cell division. Second, they dock at *lipid* *II* to create pores in the cytoplasmic membrane of the bacteria. *Class II* bacteriocins, such as sakacin, often contain amphiphilic helical structures which can insert into the cell membrane, leading to cell lysis. Bacteriolysins, such as lyostaphin, are large hydrolases which directly degrade the peptidoglycan cell wall. Reprinted with permission of Macmillan Publishers, Ltd, from Cotter et al. (2005), copyright © 2005 Nature Publishing Group

In addition to competitive exclusion of pathogens, probiotic bacteria produce numerous chemical antimicrobials which may prevent pathogenic infection. These include: hydrogen peroxide (St Amant et al. 2002; Pridmore et al. 2008), lactic acid (Fayol-Messaoudi et al. 2005), biosurfactants (Velraeds et al. 1996), immunomodulatory products (Ryan et al. 2009) and bacteriocins (Dobson et al. 2012). Bacteriocins are bacterially derived antimicrobial peptides that are active against other bacteria, but to which the producing bacterium is immune (Cotter et al. 2005). Lactic acid bacteria produce numerous broad-spectrum bacteriocins which are divided into three main classes: class I bacteriocins (lantibiotics; Schnell et al. 1988), small peptides possessing lanthionine residues; class II bacteriocins, which are heat-stable and do not contain lanthionine residues; and bacteriolysins, which are large, heat-labile murein hydrolases (Cotter et al. 2005; Fig. 5). Historically, scientists have sought to characterize the genetics and biochemistry of bacteriocins produced by LAB, in part due to their safety implications in the dairy fermentation industries (Klaenhammer 1993; Nes et al. 1996). In fact, one of the most industrially relevant bacteriocins is nisin, a lantibiotic produced by *Lactococcus lactis* (Delves-Broughton et al. 1996). Nisin has two modes of bacteriocidal activity (Fig. 5). First, it can bind lipid II, the main transporter of peptidoglycan subunits, disrupting cell wall synthesis (Breukink et al. 1999). Nisin also targets lipid II as a docking mechanism for pore formation, leading to rapid cell death due to disruption of the proton motive force (Wiedemann et al. 2001). Notably, Gram-positive bacteriocins generally have a narrow range of toxicity, as they are primarily lethal to closely related bacterial species such as *Staphylococcus*, *Listeria* and other LAB (Servin 2004). Most research involving LAB-associated bacteriocins has been in vitro. However, a landmark study by Corr et al. (2007) demonstrated that a bacteriocin produced by *L. salivarius* UCC118 caused in vivo protection in mice challenged with the food-borne pathogen *Listeria monocytogenes*. Using a functional genomics-based mutational analysis, generating a stable *L. salivarius* UCC118 strain deficient in bacteriocin production, undoubtedly established the role of this bacteriocin in protection against *L. monocytogenes* infection.

### Probiotic modulation of the gastrointestinal mucosal immune system

Perhaps one of the most important aspects of probiotic bacteria is the ability to modulate the host GIT mucosal immune system locally and systemically (Fig. 3c). The interaction between the probiotic microbe with the resident microbiota, gastrointestinal epithelia and gut immune cells to produce an immunomodulatory response is quite complex, and has been reviewed exhaustively (Lebeer et al. 2010; O’Flaherty and Klaenhammer 2010a; Reid et al. 2011; Bron et al. 2012; Klaenhammer et al. 2012; Selle and Klaenhammer 2013). Probiotic microbes modulate mucosal immunity through the interaction of proteinacious microorganism-associated molecular patterns (MAMPs) with pattern recognition receptors (PRRs) on antigen-presenting cells (APCs), such as dendritic cells and macrophages. Upon exposure to MAMPs, the PRRs (including NOD-like receptors, Toll-like receptors, and C-type lectin receptors) activate nuclear factor (NF)-κB and mitogen-activated protein kinase signaling cascades, which modulate the expression of cytokine and chemokine genes. The most common MAMPs from probiotic microorganisms are lipoteichoic acids (LTA), peptidoglycan and S-layer proteins (Bron et al. 2012). Multiple studies have explored the immunomodulatory effect of these MAMPs using functional genomic techniques. In a seminal study, the probiotics *L. casei* and *L. reuteri* were found to induce IL-10 producing regulatory T-cells through the modulation of the DC-specific ICAM-3-grabbing nonintegrin (DC-SIGN; Smits et al. 2005). Targeting of DC-SIGN by probiotic bacteria is potentially an important factor for treatment of inflammatory conditions via the production of anti-inflammatory IL-10. The S-layer protein (SlpA) of *L. acidophilus* NCFM was found to bind DC-SIGN, which regulate immature DC and T cell functionality (Konstantinov et al. 2008). Using *L. plantarum* NCIMB8826, cell wall composition was examined for immunomodulatory effects by creating a mutant (*dlt* ^−^) which produced modified teichoic acids with less d-alanine than the parent strain (Grangette et al. 2005). The mutant demonstrated a significant reduction in production of proinflammatory cytokines compared to wild type, along with a simultaneous increase in anti-inflammatory IL-10. Furthermore, the *dlt* ^−^mutant was more protective in an in vivo murine colitis model than the parent strain (Grangette et al. 2005). An LTA-deficient strain of *L. acidophilus* NCFM, created by a clean deletion of the *lba0447* phosphoglycerol transferase, was able to abate induced colonic-inflammation in a colitis mouse model through the down regulation of pro-inflammatory IL-12 and TNF-α and the up regulation of anti-inflammatory IL-10 (Mohamadzadeh et al. 2011). Additionally, this same mutant reduced colonic polyposis in a colon cancer mouse model, through the normalization of pathogenic immune responses (Khazaie et al. 2012).

Like many probiotic effectors, most MAMPs are found on the cell surface of Gram-positive microbes. Recently, the genomes and proteomes of several lactobacilli were bioinformatically screened to create a secretome database cataloging the various extracellular proteins in LAB (Kleerebezem et al. 2010; Zhou et al. 2010). Consequently, using in silico genome analysis and by reference to the LAB secretome, a putative MAMP can be selected and validated through mutagenesis (Bron et al. 2012). Indeed, a recent study of *L. acidophilus* used a proteomic-based method to identify S-layer associated proteins (SLAPs) in situ (Johnson et al. 2013). After extraction, the SLAPs were identified through mass spectrometry and referenced to the LAB secretome. Mutational analysis of one SLAP (*lba1029*), revealed an immunomodulatory phenotype using in vitro bacterial-DC co-incubation assays, suggesting the potential of multiple unknown MAMPs associated with the S-layer of *L. acidophilus* NCFM. Researchers are also trying to understand the complex dynamic of host-microbe crosstalk by using whole transcriptome profiling of human intestinal epithelia upon exposure to probiotics. In one study, transcriptomes were obtained from the mucosa of the proximal small intestines of healthy volunteers exposed to probiotic *L. acidophilus*, *L. casei*, and *L. rhamnosus* (van Baarlen et al. 2011). The transcriptional networks induced by each probiotic were unique to each strain and remarkably similar to response profiles obtained from bioactive components and drug treatments. In vitro transcriptome profiling of Caco-2 intestinal epithelial cell lines exposed to *L. acidophilus* NCFM corroborated these data (O’Flaherty and Klaenhammer 2012). Similarly, *Bifidobacterium bifidum* PRL2010 transcriptome analyses with both in vitro human cell lines and in vivo murine models demonstrated the capacity for strain PRL2010 to modulate host innate immunity (Turroni et al. 2014).

## Conclusions and future directions

While the paradigm of discovery based genomics in probiotic LAB has uncovered vital aspects of probiotic mechanisms, it has also revealed the complexity of these interactions with the resident microbiota and the mucosal immune system. But with this challenge has come great opportunity. For example, probiotic bacteria are now being explored as suitable models for vaccine/drug delivery, due to their close association with host immunity and immunomodulatory action (Kajikawa et al. 2011; Stoeker et al. 2011; Kajikawa et al. 2012). Furthermore, recent discoveries are also demonstrating that the roles of probiotic bacteria and the resident microbiota extend far beyond gastrointestinal health. Specifically, studies on the bi-directional crosstalk between the GIT and the brain (the gut-brain axis) are revealing the neurochemical importance of gut homeostasis (Cryan and Mahony 2011; Bercik et al. 2012). Along with these advancements, it is important that human clinical trials continue with experimental designs that are well-controlled and well-defined, reflecting the great progress that has been made in the field of probiotic and GIT microbiome research (reviewed by Sanders et al. 2013). With more than a century passing since Metchnikoff’s observations, keen experimental design using integrated genomics has led to a clearer definition of probiotic bacteria, as well as a model for continued discovery.
